# MDH2 regulates the sensitivity of clear cell renal cell carcinoma to ferroptosis through its interaction with FSP1

**DOI:** 10.1038/s41420-024-02137-6

**Published:** 2024-08-13

**Authors:** Baijie Feng, Wei Su, Xianzhi Guo, Tingting Ding, Yingchun Duan, Lina Hu, Minghua Yu

**Affiliations:** 1 Fudan University Clinical Research Center for Cell-based Immunotherapy & Department of Oncology, Fudan University Pudong Medical Center, Shanghai, P. R. China; 2 Department of Medical Oncology, Fudan University Shanghai Cancer Center, Shanghai, China; 3 Department of Gynecology and Obstetrics, Shanghai Pudong Hospital, Fudan University Pudong Medical Center, Shanghai, P. R. China

**Keywords:** Cancer therapy, Cell death

## Abstract

Malate dehydrogenase 2 is a pivotal enzyme in the tricarboxylic acid cycle. Recent studies have highlighted the significant involvement of MDH2 in the pathogenesis and progression of diverse types of tumors, yet its precise mechanistic underpinnings remain elusive. This study revealed a significant decrease in MDH2 expression in renal cancer tissues. And knocking out MDH2 was observed to hinder the proliferation of normal renal tubular epithelial cells but notably enhance the proliferation of ccRCC. Furthermore, mechanistically, we found that MDH2 inhibits the proliferation of ccRCC by promoting ferroptosis, while enhancing the sensitivity of ccRCC to ferroptosis inducers, promoting lipid peroxidation. We also demonstrated that MDH2 regulates the ubiquitination of FSP1 through protein-protein interactions, leading to a decrease in FSP1 protein levels and maintaining high sensitivity of ccRCC to ferroptosis. In conclusion, our study demonstrates that the reduced MDH2 expression in ccRCC results in increased expression of FSP1, thereby reducing its sensitivity to ferroptosis. It unveils a non-metabolic role for the downregulation of MDH2 in ccRCC progression.

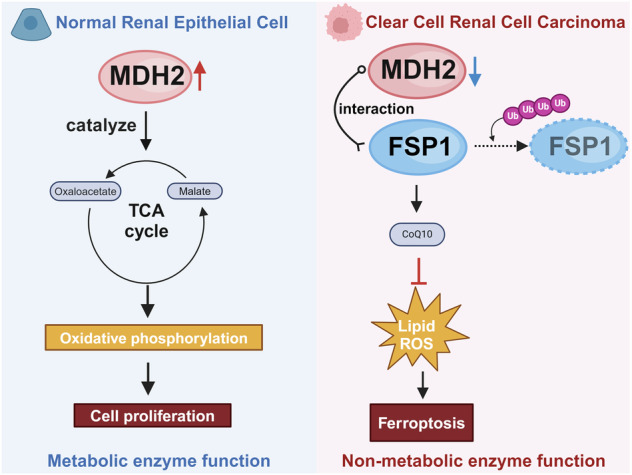

## Introduction

Renal cell carcinoma (RCC) is a malignant tumor that originates in the kidney and has a high mortality rate [1]. Clear cell renal cell carcinoma (ccRCC) is the most common histological subtype of RCC, accounting for approximately 75% of cases [2, 3]. The main treatment modalities for ccRCC currently include surgery [4], intervention, targeted therapy, and immunotherapy [5, 6]. Despite recent advances in treatment modalities, advanced ccRCC is a generally incurable malignancy with a high mortality rate. There is an urgent need for novel biologic insights in this disease to further advance the treatment landscape [7].

Malate dehydrogenase 2 (MDH2) is a pivotal enzyme in the tricarboxylic acid (TCA) cycle, facilitating the reversible conversion of malate to oxaloacetate with NAD^+^ as a cofactor [8]. Analysis of the Cancer Genome Atlas (TCGA) databases revealed notable variations in MDH2 expression levels between ccRCC and normal kidney tissue. MDH2 is intricately associated with maintaining normal cellular function and the pathogenesis of various diseases. The OAA produced by MDH2 rewires the fueling of the respiratory chain and the TCA cycle [9], MDH2 promotes the growth of ovarian cancer by activating mitochondrial respiration [10]. While existing research predominantly emphasizes the metabolic function of MDH2, scant attention has been paid to its non-metabolic functions. Consequently, our objective is to explore the potential non-metabolic enzyme function of MDH2 in ccRCC.

Ferroptosis is a novel type of cell death that differs from apoptosis, necrosis, or autophagic cell death [11]. In recent years, extensive research has shown that ferroptosis is the result of iron-dependent lipid peroxidation [12, 13]. Many studies have found a correlation between ferroptosis and tumor [14]. Inducing ferroptosis has become an attractive strategy for treating various types of cancer [15, 16]. Ferroptosis suppressor protein 1 (FSP1, also known as apoptosis-inducing factor mitochondrial 2, AIFM2), plays a key role in regulating ferroptosis [17, 18]. In particular, when FSP1 is localized to the cell membrane, it acts as an oxidoreductase, reducing coenzyme Q10 (CoQ10) to generate a lipophilic radical-trapping antioxidant, thus halting the propagation of lipid peroxides [17]. It is worth noting that the expression levels of various ferroptosis-related genes are significantly correlated with the occurrence and development of ccRCC patients [7], indicating that ferroptosis plays a crucial role in the progression of ccRCC [19].

The present study reveals a significant downregulation of MDH2 expression in ccRCC tumor tissues. Furthermore, gene editing techniques were employed to manipulate the expression of MDH2, enabling investigation into its role in ccRCC cell proliferation and regulation of ferroptosis sensitivity. Finally, we conducted an in-depth investigation into the precise mechanisms underlying the impact of MDH2 on tumor growth and its influence on ferroptosis sensitivity in ccRCC. Our study elucidates that the interaction between MDH2 and FSP1 regulates susceptibility to ferroptosis in ccRCC, thereby providing novel insights into the potential role of common metabolic enzymes in regulating tumorigenesis and tumor progression.

## Result

### MDH2 is downregulated in ccRCC

**Fig. 1:**
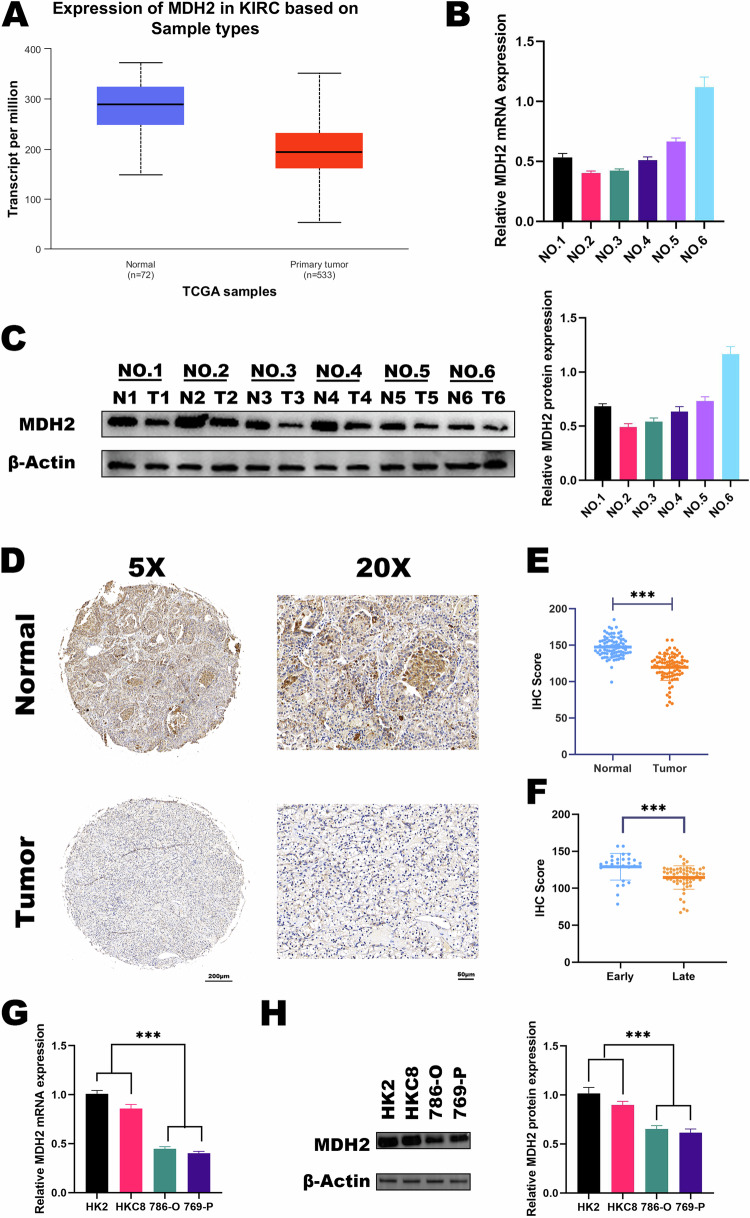
MDH2 is downregulated in ccRCC and associated with its staging. **A** Expression of MDH2 in ccRCC and normal renal tissue according to TCGA database. **B** The mRNA level of *MDH2* in ccRCC and adjacent normal tissues (*n* = 6). The expression level of *β*-Actin was used as normalized control. The statistical analysis comes from technical replicates and the experiment was repeated three times. Data was presented as mean ± SD. **C** The protein level of *MDH2* and *β*-Actin in ccRCC and adjacent normal tissues (*n* = 6). The statistical analysis comes from technical replicates and the experiment was repeated three times. (T tumor tissue, N normal tissue). Data was presented as mean ± SD. **D** Representative images of MDH2 staining in ccRCC tumor tissues and normal tissues. **E** The IHC score of *MDH2* in normal tissues and ccRCC tissues. **F** The IHC score of *MDH2* in different histological grades of ccRCC tissues. **G** The *MDH2* mRNA level in the normal kidney cell lines (HK2 and HKC8) and ccRCC cell lines (786-O and 769-P). The expression level of *β*-Actin was used as the normalized control. The statistical analysis comes from biological replicates and the experiment was repeated three times. Data was presented as mean ± SD. **H** The protein level of MDH2 in the normal kidney cell line (HK2 and HKC8) and ccRCC cell lines (786-O and 769-P), and *β*-Actin was used as control. The statistical analysis comes from biological replicates and the experiment was repeated three times. Data was presented as mean ± SD. (*P < 0.05, **P < 0.01, ***P < 0.001, and ns not significant.).

To investigate the role of MDH2 in ccRCC, we analyzed the TCGA datasets. The results showed a significant decrease in the expression of MDH2 in tumor tissues compared to normal tissues (Fig. 1A).

Additionally, we obtained a collection of renal cell carcinoma tissues along with their corresponding normal tissues to extract RNA and proteins for reverse transcription and quantitative PCR (RT-qPCR) and WB detection. The results demonstrated that the levels of MDH2 mRNA and protein were lower in ccRCC tumor tissues compared to normal tissues (Fig. 1B, C).

To further investigate the expression of MDH2 in ccRCC, we assessed the expression of MDH2 in tumor tissues of the kidney and the adjacent non-tumor tissues of 85 patients diagnosed with ccRCC (Table 1) using immunohistochemical (IHC) staining analysis. We observed varying intensities of MDH2 staining in both tumor tissues and their corresponding adjacent non-tumor tissues. Moreover, we observed a significant downregulation of MDH2 expression in ccRCC tissues as compared to their adjacent non-tumor tissues (Fig. 1D, E). Meanwhile, we have found that MDH2 is associated with the staging of ccRCC, while MDH2 has a higher expression in the early stages (Fig. 1G). The summarized clinical characteristics of the patients are as follows: Supplementary Table 1. At the same time, we compared the expression of MDH2 in immortalized human kidney cell lines and ccRCC cell lines. The results showed that compared to immortalized human kidney cell lines, both mRNA and protein expression of MDH2 were decreased in the ccRCC cell lines.

**Table 1:** The clinicopathological characteristics of 85 ccRCC patients.

Characteristic	Patient
All patient	85
Gender	
Male	37
Female	48
Age (years)	
≤55	14
>55	71
Stage	
I+II	29
III+IV	56
T stage	
T1 + T2	26
T3 + T4	59
N stage	
N0	23
N1	62
M stage	
M0	43
M1	42
Tumor size (cm)	
≤4	21
>4	64

In conclusion, these findings implied MDH2 is downregulated in ccRCC and is correlated with staging.

### MDH2 regulates the proliferation of ccRCC cells through ferroptosis

**Fig. 2:**
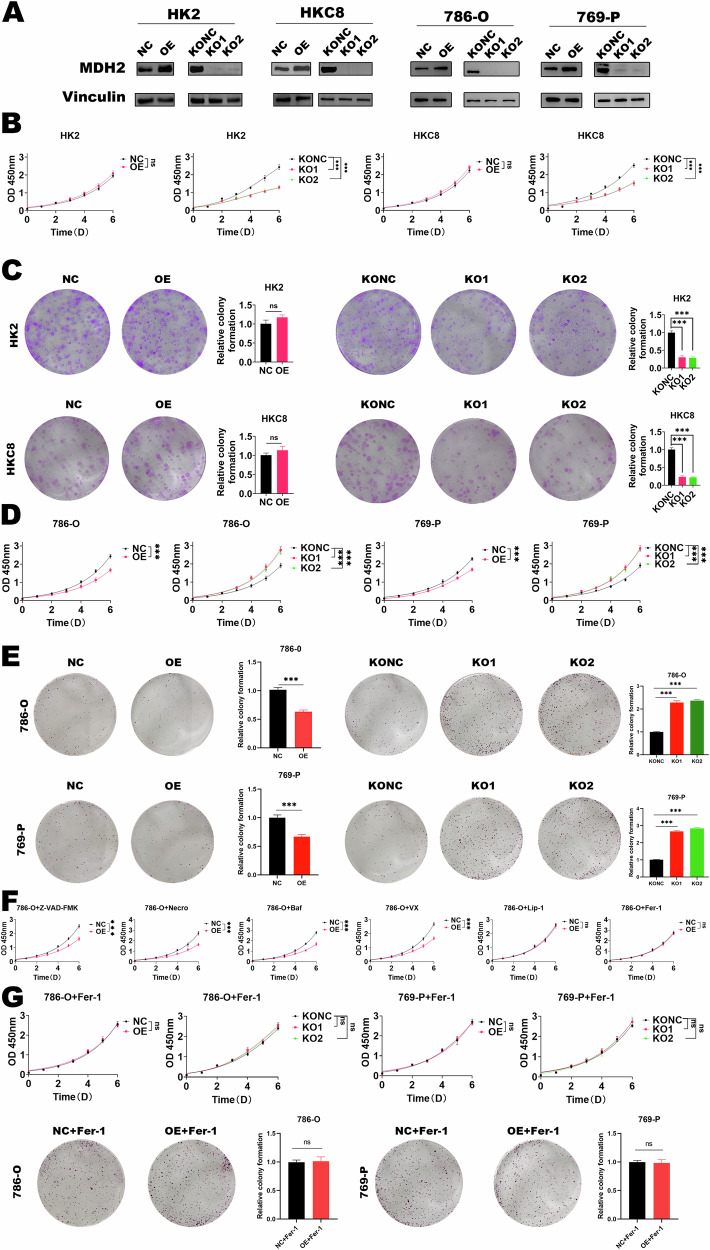
MDH2 regulates ccRCC proliferation through ferroptosis. **A** Validation of MDH2 overexpression and knockout cell lines by Western blotting (NC: negative control for overexpression, OE: overexpression, KONC: negative control for knockout, KO1/KO2: knockout cell lines.). **B** MDH2 regulates the proliferation ability of normal renal epithelial cell lines. The statistical analysis comes from biological replicates and the experiment was repeated three times. Data was presented as mean ± SD. **C** MDH2 regulates the colony formation ability of normal renal epithelial cell lines. 50 visible colonies were observed on the 14th day for HK2 and HKC8. The statistical analysis comes from biological replicates and the experiment was repeated three times. Data was presented as mean ± SD. **D** MDH2 suppressed cell growth ability in ccRCC cells. The statistical analysis comes from biological replicates and the experiment was repeated three times. Data was presented as mean ± SD. **E** MDH2 suppressed colony formation ability in ccRCC. 50 visible colonies were observed on the 9th day for 786-O; on the 10th day for 769-P. The statistical analysis comes from biological replicates and the experiment was repeated three times. Data was presented as mean ± SD. **F** Treatment with regulated cell death inhibitors, including z-VAD-FMK (10 μmol/L), necrostatin-1 (20 μmol/L), bafilomycin A1 (100 nmol/L), VX-765 (5 μmol/L), liproxsrain-1 (10 μmol/L), and ferrostatin-1 (5 μmol/L), in 786-O MDH2-OE cells, and cell growth ability was evaluated by CCK-8. The statistical analysis comes from biological replicates and the experiment was repeated three times. Data was presented as mean ± SD. **G** ccRCC cells with 5 μmol/L ferrostatin-1, and cell growth ability and colony formation ability were evaluated. The statistical analysis comes from biological replicates and the experiment was repeated three times. Data was presented as mean ± SD. (*P < 0.05, **P < 0.01, ***P < 0.001, and ns not significant.).

We constructed cell lines overexpressing and knocking out MDH2 in immortalized renal epithelial cells and ccRCC cell lines, respectively (Fig. 2A). We found that knocking out MDH2 significantly impaired the proliferation (Fig. 2B) and colony formation ability of renal epithelial cells (Fig. 2C), while overexpression of MDH2 did not show substantial changes. This may be because knocking out MDH2 damages the TCA cycle. However, in ccRCC cell lines, significantly different results were observed. Knocking out MDH2 significantly enhanced their proliferation (Fig. 2D) and colony formation ability (Fig. 2E), while overexpression of MDH2 instead slowed down their proliferation. MDH2 plays the specificity of functional effects in ccRCC cells.

In order to investigate the relationship between MDH2 regulation of ccRCC proliferation and programmed cell death, we used various programmed cell death inhibitors to intervene in MDH2-OE and NC cell lines. We found that only the ferroptosis inhibitor could restore the proliferation slowdown induced by MDH2 overexpression (Fig. 2F). Furthermore, this conclusion was further validated in MDH2 knockout cell lines (Fig. 2G).

### MDH2 promotes sensitivity to ferroptosis in ccRCC

**Fig. 3:**
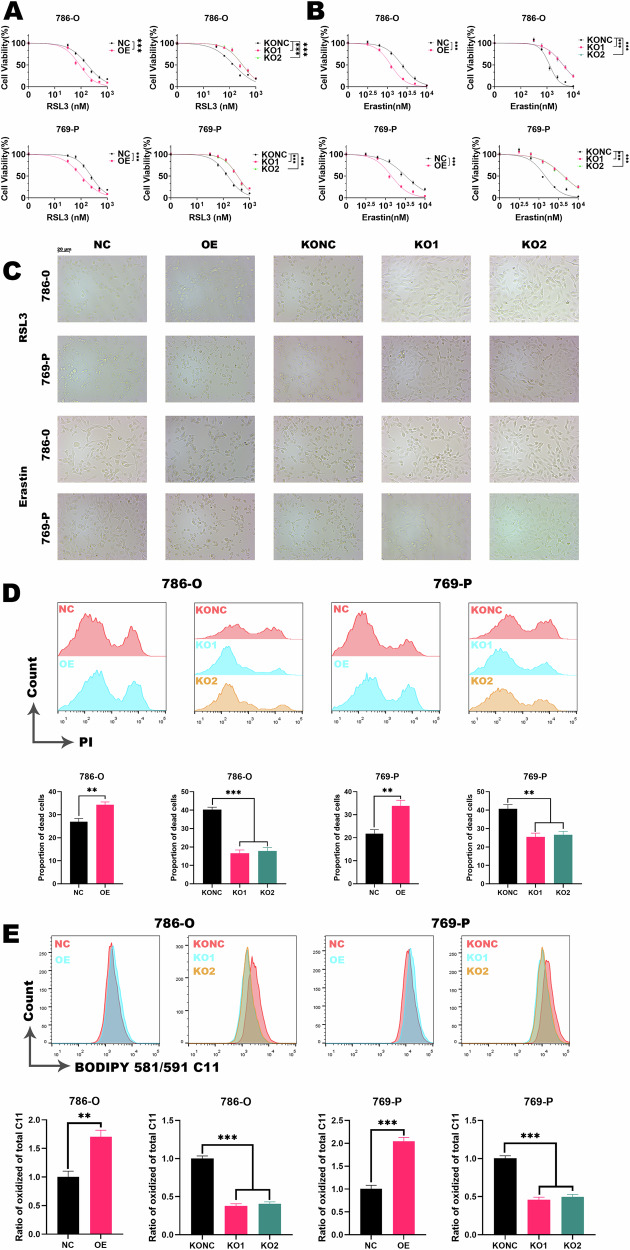
MDH2 enhances the sensitivity of ccRCC to ferroptosis. **A**, **B** Response curves of ccRCC cells to RSL3 (**A**) and erastin (**B**). The statistical analysis comes from biological replicates and the experiment was repeated three times. Data was presented as mean ± SD. **C** Morphology of ccRCC cells treated with RSL3 (0.2 μM, 6 h). **D** Flow cytometry analysis of cell death in ccRCC cells treated with RSL3 (0.5 μM, 6 h). The statistical analysis comes from technical replicates and the experiment was repeated three times. Data was presented as mean ± SD. **E** Flow cytometry analysis of lipid peroxidation in ccRCC cells treated with RSL3 (0.1 μM, 6 h). The statistical analysis comes from technical replicates and the experiment was repeated three times. Data was presented as mean ± SD. (*P < 0.05, **P < 0.01, ***P < 0.001, and ns not significant.).

In order to further investigate the relevance of MDH2 and ferroptosis in ccRCC, we used common ferroptosis inducers (RSL3, erastin) to intervene in cell lines. The results indicate that the knockout of MDH2 significantly confers resistance to the decrease in cell viability induced by ferroptosis inducers (Fig. 3A, B). And the changes in cell morphology after treatment with ferroptosis inducers were observed (Fig. 3C). We found that knockout of MDH2 conferred resistance to ferroptosis in renal cancer cells, and reduced morphological changes. Additionally, we found that MDH2 knockout led to decreased cell death (Fig. 3D) and reduced lipid peroxidation (Fig. 3E) in ccRCC cells induced by RSL3. Moreover, opposite effects were observed in renal cancer cells with stable overexpression of MDH2. These results suggest that MDH2 significantly promotes the sensitivity of ccRCC cells to ferroptosis.

### MDH2 regulates ccRCC ferroptosis sensitivity by modulating FSP1

**Fig. 4:**
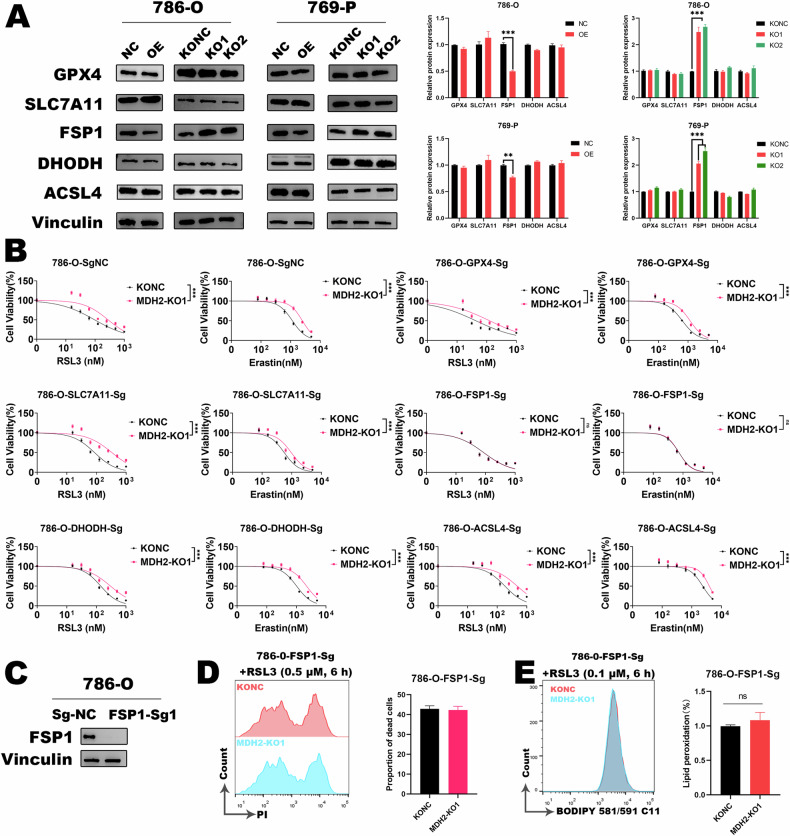
MDH2 regulates the sensitivity of ferroptosis through the modulation of FSP1. **A** Protein expression levels of in ferroptosis key factor in MDH2 overexpressing and knockout ccRCC cells. The statistical analysis comes from biological replicates and the experiment was repeated three times. Data was presented as mean ± SD. **B** Response-dose curves of dual-knockout cells to RSL3 and erastin. The statistical analysis comes from biological replicates and the experiment was repeated three times. Data was presented as mean ± SD. **C** WB assay detected the knockout efficiency of FSP1. **D** Flow cytometry analysis of cell death in FSP1/MDH2 dual-knockout cells treated with RSL3 (0.5 μM, 6 h). The statistical analysis comes from technical replicates and the experiment was repeated three times. Data was presented as mean ± SD. **E** Flow cytometry analysis of lipid peroxidation in FSP1/MDH2 dual-knockout cells treated with RSL3 (0.1 μM, 6 h). The statistical analysis comes from technical replicates and the experiment was repeated three times. Data was presented as mean ± SD. (*P < 0.05, **P < 0.01, ***P < 0.001, and ns not significant).

To further explore the mechanisms by which MDH2 regulates sensitivity of ferroptosis in ccRCC, we examined the protein expression levels of known key molecules involved ferroptosis. Western blot revealed that the protein expression of FSP1 were significantly upregulated in 786-O and 769-P cells upon MDH2 knockout (Fig. 4A). However, the expression of other key proteins, such as GPX4, SLC7A11, ACSL4, and DHODH, showed no significant changes (Fig. 4A). Similar conclusions were obtained in overexpression cell lines. Based on these data, we hypothesize that FSP1 may be an important downstream molecule regulated by MDH2 in ferroptosis.

To further confirm that MDH2 regulates ccRCC ferroptosis sensitivity through FSP1, we engineered knockout cell lines of various key ferroptosis genes including FSP1 (Fig. 4B, C). Subsequently, we further knocked out MDH2 on this basis to generate double-knockout cell lines.

We found that the knockout of MDH2 no longer affects the sensitivity of ccRCC cells to ferroptosis in the background of FSP1 knockout (Fig. 4B). However, in the background of knockout of other key ferroptosis genes, the knockout of MDH2 still leads to significant resistance of ccRCC cells to ferroptosis (Fig. 4B). We further utilized flow cytometry to detect cell death and lipid peroxidation. In the background of FSP1 knockout, we observed that the knockout of MDH2 similarly does not result in a reduction in the proportion of cell death (Fig. 4D) or the level of lipid peroxidation (Fig. 4E). In conclusion, our results indicate that MDH2 regulates the sensitivity of ferroptosis through FSP1.

### MDH2 interacts with FSP1 and promotes its ubiquitination

**Fig. 5:**
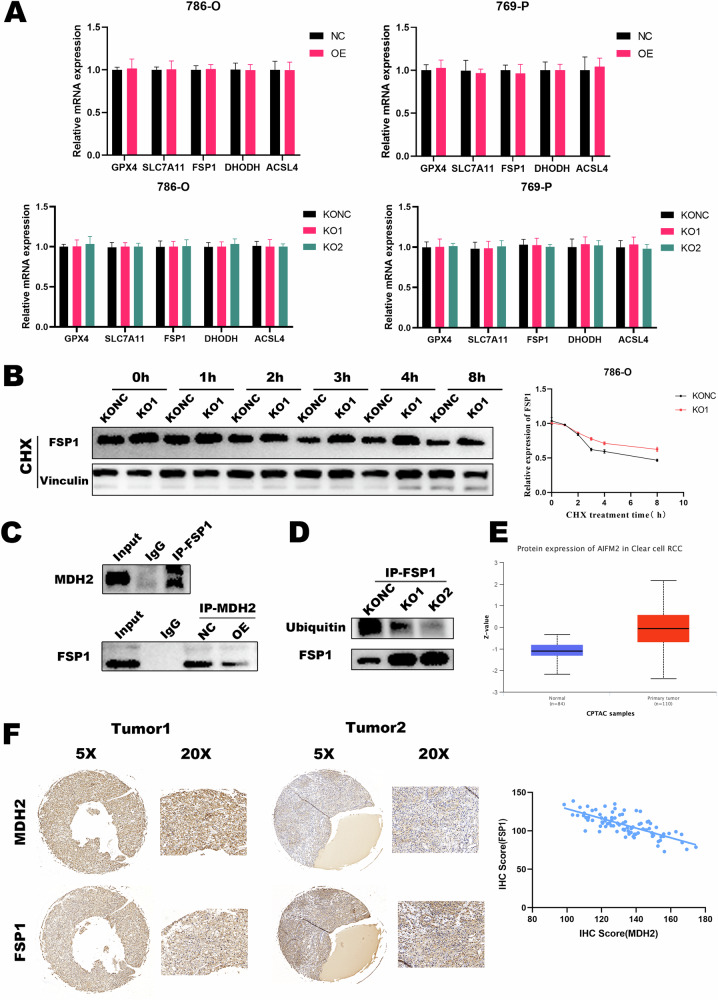
MDH2 interacts with FSP1 and promotes its ubiquitination. **A** mRNA expression levels of ferroptosis key gene in MDH2 overexpressing and knockout ccRCC cells. The expression level of *β*-Actin was used as normalized control. The statistical analysis comes from biological replicates and the experiment was repeated three times. Data was presented as mean ± SD. **B** Western blot was performed to detect the stability of FSP1 protein in MDH2 knockout and negative control cells. The statistical analysis comes from biological replicates and the experiment was repeated three times. Data was presented as mean ± SD. **C** Co-IP detecting the interaction between MDH2 and FSP1 in 786-O cells. **D** WB detection of FSP1 ubiquitination in 786-O cells. The statistical analysis comes from biological replicates and the experiment was repeated three times. Data was presented as mean ± SD. **E** Protein expression of FSP1 from the CPTAC ccRCC dataset. **F** Investigate the correlation between MDH2 and FSP1 in tissue samples from ccRCC patients. (*P < 0.05, **P < 0.01, ***P < 0.001, and ns not significant.).

In order to elucidate the mechanism by which MDH2 regulates the expression of FSP1, we employed the RT-qPCR method to detect the expression of FSP1 mRNA at the transcriptional level (Fig. 5A). Interestingly, there was no significant difference in the mRNA levels of FSP1 in cells overexpressing or knockout for MDH2. Therefore, we hypothesize that MDH2 may regulate the expression of FSP1 at the protein level. After further intervention with CHX and MG132 in MDH2 knockout cells and their controls, we observed the time-dependent changes in the expression level of the FSP1 protein. It was found that MDH2 mainly influences the degradation rate of FSP1 rather than its generation rate (Fig. 5B). Considering that both MDH2 and FSP1 are located in the mitochondria, we investigated whether MDH2 interacts directly with FSP1. As expected, Co-immunoprecipitation (Co-IP) confirmed the direct interaction between MDH2 and FSP1 (Fig. 5C). Further investigation revealed that MDH2 can regulate the ubiquitination of FSP1, promoting its degradation and thereby reducing the protein expression of FSP1 (Fig. 5D). Taken together, these data demonstrate the interaction between MDH2 and FSP1, and their involvement in the ubiquitination and degradation of FSP1.

From the data of the CPTAC ccRCC dataset, we found a significantly higher protein expression of FSP1/AIFM2 in ccRCC compared to normal kidney (Fig. 5E), which is in contrast to MDH2. Moreover, we conducted an analysis on the correlation between MDH2 and FSP1 using a dataset of 85 tissue samples collected from patients with ccRCC. The results revealed a significant negative correlation between MDH2 and FSP1 in ccRCC, with tissues affected by ccRCC demonstrating a tendency for higher MDH2 expression and lower expression of FSP1 (Fig. 5F).

### Knocking out MDH2 promotes the proliferation of ccRCC cells and inhibits the sensitivity to ferroptosis in vivo

**Fig. 6:**
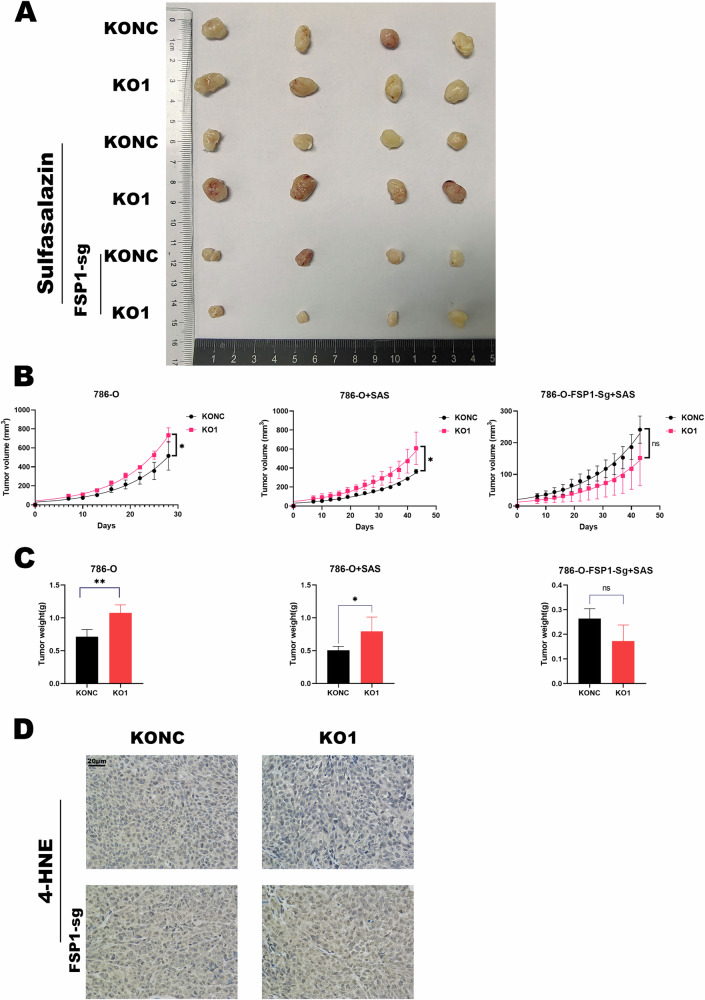
MDH2 regulates the proliferative ability and sensitivity to ferroptosis of ccRCC in vivo. **A**–**C** Representative images (**A**), growth curves (**B**), and tumor weight (C) of subcutaneous tumors were assessed in each cohort of nude mice. **D** Immunohistochemistry detection of 4-HNE from xenografts. (SAS: 250 mg/kg/day.) Data are presented as representative images or as the mean ± SD of four independent experiments. (*P < 0.05, **P < 0.01, ***P < 0.001, and ns not significant.).

We established a model of subcutaneous thymus-free mouse tumors by injecting cells with MDH2 knockout and control into the subcutaneous area of nude mice. It showed that compared to the NC group, the MDH2 knockout group had a significantly faster tumor growth rate, as well as increased tumor weight and volume (Fig. 6A–C). These results indicate that knocking out MDH2 can promote the growth of ccRCC cells under in vivo conditions. Additionally, when we treated the MDH2 knockout group with the ferroptosis inducer SAS, the tumor volume and weight were still significantly increased compared with the NC group (Fig. 6A–C). This suggests that MDH2 inhibits ccRCC proliferation while promoting their sensitivity to ferroptosis.

Subsequently, we employed 4-HNE to detect subcutaneous tumors in four groups of mice that were injected with SAS. The results demonstrated a decrease in 4-HNE expression in the MDH2 KO1 group compared to the KONC group (Fig. 6D). Furthermore, no significant difference in the expression of 4-HNE was observed between the two groups in the presence of FSP1-Sg (Fig. 6D). Collectively, these findings indicated that MDH2 modulates the expression of FSP1, thereby regulating the sensitivity of ccRCC to ferroptosis.

## Discussion

Abnormal proliferation and resistance to programmed cell death are the most common characteristic changes in cancers [20]. In this study, we focused on MDH2, one of the key enzymes in the TCA cycle, and found its unique non-metabolic function in ccRCC.

Through the analysis of TCGA database and clinical samples, we found that MDH2 is significantly downregulated in ccRCC compared to normal kidney tissue and is associated with its staging. In further experiments, we constructed cell lines overexpressing and knocking out MDH2 in normal renal epithelial cell lines and ccRCC cell lines respectively, and discovered an interesting phenomenon. In normal renal epithelial cells, knocking out MDH2 significantly inhibits proliferation, while overexpressing MDH2 shows no significant change. In ccRCC, knocking out MDH2 significantly promotes proliferation, whereas overexpressing MDH2 inhibits proliferation. This difference may be caused by the different dependency levels on the TCA cycle between normal cells and tumor cells. The energy demand of normal renal tubular cells depends on TCA cycle and oxidative phosphorylation [21]. The kidney is particularly susceptible to the dysfunction of TCA cycle, so disrupting the integrity of the TCA cycle will severely impede normal kidney function [22]. Cancer cells exhibit a high rate of glucose consumption, generating ATP through glycolysis and shifting the energy source from mitochondrial oxidative phosphorylation to glycolysis [23, 24]. They are less affected by TCA cycle dysfunction [25, 26]. Previous studies have found significantly lower levels of malate in ccRCC [27], and mutations in MDH2 do not lead to accumulation of large amounts of malate [28]. Therefore, the requirement of MDH2 functioning as metabolic enzyme is greatly reduced in ccRCC. However, the abnormal over-proliferation of ccRCC after knocking out MDH2 is confusing, so we hypothesize that besides serving as a catalytic enzyme, MDH2 also has some unknown functions.

In further studies, the changes in proliferative capacity induced by MDH2 in ccRCC can be restored by ferroptosis inhibitors. This result indicates that MDH2 may have a function in regulating the sensitivity of ccRCC to ferroptosis. We used a common ferroptosis inducer to intervene and found that knockouting MDH2 significantly inhibited lipid peroxidation and ferroptosis, while overexpressing MDH2 promoted ferroptosis. Ferroptosis is a programmed form of cell death distinct from apoptosis, primarily caused by excessive lipid peroxidation [11]. Ferroptosis plays a significant role in the occurrence, development, invasion, metastasis, and resistance to treatment in various tumors [16, 19]. The sensitivity of cells to ferroptosis is mainly mediated by the ferroptosis defense system, lipid metabolism, and iron metabolism [13]. We detected various key targets regulating the sensitivity to ferroptosis and found the protein expression of FSP1 was significant changed. In order to further validate the mechanism of MDH2 mediating the sensitivity of ccRCC to ferroptosis, we constructed knockout cell lines of various key targets of ferroptosis and then knocked out MDH2 on this basis, ultimately establishing double-knockout cell lines. We found that the function of MDH2 mediating ferroptosis depends on the expression of FSP1, rather than other targets. FSP1 was initially identified as an apoptosis-related protein and named AIFM2 [17]. FSP1/CoQ10 defense system is parallel to SLC7A11/GSH/GPX4 defense system and can protect cells from ferroptosis in the absence of GPX4 [18]. Due to its discovery of an important role in ferroptosis in 2019, it was suggested to be renamed as FSP1. In recent years, research on FSP1 has rapidly increased [29], but no reports on the relationship between MDH2 and FSP1 have been seen yet.

FSP1 expression is significantly elevated in ccRCC compared to normal kidney tissue, and it can promote the occurrence and development of ccRCC by enhancing the ferroptosis defense system. In order to further investigate the mechanism of MDH2 regulating the FSP1/CoQ10 defense system, we detected the changes in the mRNA expression of FSP1 and found that it did not alter the transcription level of FSP1. Due to the regulation of FSP1 protein expression by MDH2, we measured the degradation rate of FSP1 and found that knocking out MDH2 significantly slowed down the degradation of FSP1. Subsequent Co-IP experiments showed the existence of protein-level interaction between MDH2 and FSP1, and this interaction significantly promoted FSP1 ubiquitination. This has not been reported in previous studies and represents another non-metabolic function of MDH2 apart from catalyzing the TCA cycle.

In conclusion, our study demonstrates that the reduced MDH2 expression in ccRCC results in increased expression of FSP1, through decreases ubiquitination of FSP1 by interacting with FSP1, and ultimately enhances the ferroptosis defense system in ccRCC. It unveils a non-metabolic role for the downregulation of MDH2 in ccRCC progression.

## Material and methods

### TCGA database and related analysis tools

TCGA-Kidney renal clear cell carcinoma (TCGA-ccRCC, https://cancergenome.nih.gov/) contains 533 cases of renal cancer and 72 cases of normal control. These cases provide basic information such as age, gender, race, medical history, diagnosis type, and tumor staging. The CPTAC ccRCC dataset consists of 110 cases of ccRCC and 84 cases of normal control. Gene and protein expression analysis as well as visualization were conducted using the UALCAN website tool (http://ualcan.path.uab.edu/) [30].

### Patients and tissue samples

We collected tumor and normal tissue samples from 85 ccRCC patients (Table 1) and performed IHC to analyze the expression of MDH2 and FSP1 in both normal and ccRCC tumor tissues. Tissue samples obtained from ccRCC patients who underwent nephrectomy were utilized for RNA and protein extraction, followed by subsequent analysis. Written consent was obtained from all patients for sample collection. This study has received approval from the Ethics and Research Committee of Shanghai Pudong Hospital.

### Reagents

Erastin (HY-15763), Z-VAD-FMK (HY-16658B), Necrostatin-1 (HY-15760), Bafilomycin A1 (HY-100558), VX-765 (HY-13205), Liproxstatin-1 (HY-12726) were purchased from MedChemExpress (Shanghai, China). Ferrostain-1 (S7243) and RSL3 (S2767) were purchased from Selleck Chemical (Shanghai, China).

### Cell lines and cell culture

The HK2, HKC8, 786-O and 769-P RCC cell lines were purchased from the Cell Bank of the Chinese Academy of Sciences (Shanghai, China). HK2 and HKC8 were cultured in DMEM/F12 medium (Hyclone, GE Healthcare Life Sciences, Logan, UT, USA) supplemented with 10% FBS (Biological Industries) at 37 °C with 5% CO_2_. 786-O and 769-P were cultured in RPMI 1640 medium (Hyclone, GE Healthcare Life Sciences, Logan, UT, USA) supplemented with 10% FBS at 37 °C with 5% CO_2_. All cells have recently undergone STR profiling identification and excluded mycoplasma contamination.

### Construction of MDH2 overexpression and knockout cell lines

The PLVX-MDH2 plasmids were purchased from HedgehogBio, Inc. (Shanghai, China). The sgRNAs (KO1: TATCGCGCACACACCCGGAG; KO2: GCGTGTACCTGAGAGATCAG) of human MDH2 were synthesized by Genomeditech, Inc. (Shanghai, China) and cloned into the LentiGuide-Puro to construct the LentiGuide-MDH2-Puro knockout plasmids. Subsequently, the plasmids were co-transfected into HEK293T cells with Lipofectamine 3000 (Invitrogen, Inc.) along with psPAX2 and PMG.2 G plasmids to generate lentivirus. Then, HK2, HKC8, 786-O and 769-P cells were infected with the lentivirus (multiplicity of infection, MOI = 5) and incubated for 72 h. After 72 h of screening with 2 μg/mL of puromycin, single cells were sorted into 96-well plates to obtain single clones. Finally, the clones were validated by Western blot analysis to obtain overexpression and knockout of MDH2 cells.

### Construction of dual-gene knockout cell lines

The sgRNAs of GPX4, SLC7A11, AFIM2, DHODH and ACSL4 were synthesized by Genomeditech, Inc. (Shanghai, China) and cloned into the LentiGuide-Hygro to construct the LentiGuide-Hygro knockout plasmids. Afterwards, the same method was used to package the lentivirus and infect cells. After 1-2 weeks of hygromycin screening, the corresponding gene knockout cell lines were obtained through monoclonal screening. Finally, the clones were validated by Western blot analysis. On this basis, LentiGuide-MDH2-Puro virus was infected again, and after screening using the same method as mentioned above, dual-gene knockout cells were constructed.

### Cell proliferation assay

500 cells were seeded in each well of a 96-well plate and CCK-8 experiments were performed daily for the following 6 days. 10 µl of Cell Counting Kit-8 reagent (CCK-8; Dojindo Molecular Technologies, Japan) was added to the wells to be tested, and the absorbance at a wavelength of 450 nm was measured after incubating for 2 h.

### Clonogenic assay

10^3^ cells were seeded in each well of a 6-well plate and cultured at 37 °C. Once the number of visible colonies reached 50 cells, they were fixed. Polyformaldehyde was used for fixation for 15 min, followed by staining with 0.2% crystal violet for 30 min. Finally, the cells were washed three times with PBS. 50 visible colonies were observed on the 9th day for 786-0; on the 10th day for 769-P; on the 14th day for HK2; on the 14th day for HKC8.

### Cell viability assay

5000 cells were seeded in each well of a 96-well plate. After 24 h of incubation, different concentrations of RSL3 and erastin were added, followed by an additional incubation of 6 h and 24 h respectively. Then, 10 µl of Cell Counting Kit-8 reagent (CCK-8; Dojindo Molecular Technologies, Japan) was added to each well and incubated for 2 h. Subsequently, the absorbance of each well was measured at a wavelength of 450 nm using an enzyme-linked immunosorbent assay reader.

### Flow cytometry for detecting cell death and lipid peroxidation

10^6^ cells were seeded in each well of a 6-well plate and cultured for 24 h. Then, the cells were treated with the desired concentrations of RSL3 and erastin for 6 h and 24 h, respectively. For cell death detection, the cells were incubated with 1 μg/mL propidium iodide (PI) (Invitrogen) in PBS, while for lipid peroxidation detection, the cells were incubated with 2 μM C11 BODIPY 581/591 (Invitrogen, Carlsbad, CA, USA). After 30 min of light avoidance incubation, the cells were washed twice with PBS, collected, and analyzed using an Accuri C6 flow cytometer (BD Biosciences, San Jose, CA, USA).

### Light microscopy

5 × 10^5^ cells were seeded in each well of a 6-well plate and cultured for 24 h. Then, the cells were treated with the desired concentrations of RSL3 and erastin for 6 h and 24 h, respectively. Subsequently, phase-contrast images of the cells were obtained using an inverted phase-contrast microscope (Olympus) at a magnification of 200x.

### Western blotting

10^6^ cells were seeded in a 6-well plate and allowed to adhere. After that, an appropriate amount of RIPA lysis buffer was added, along with 1% protease inhibitor and phosphatase inhibitor. The cells were lysed on ice for 15 min and then centrifuged to collect the supernatant. The protein concentration was determined using a BCA protein assay kit (Thermo Fisher Scientific, USA). A suitable concentration of SDS-PAGE gel was prepared, and the proteins were electrophoresed and transferred onto a PVDF membrane (0.2μm/0.45μm, Solarbio, Beijing, China). The membrane was blocked with a protein-free blocking solution, followed by cutting out the corresponding bands and incubating with primary antibodies overnight at 4°C. After washing with TBST three times, the appropriate secondary antibodies were incubated at room temperature for 1 h. The membrane was then washed again with TBST. Finally, the protein bands were visualized using an ECL reagent (Thermo Fisher Scientific, MA, USA). The following antibodies were used: GPX4, SLC7A11, DHODH, ACSL4, Vinculin (1:1000, Abclonal, China), MDH2, FSP1 (1:1000, Proteintech Group, Inc.), Ubiquitin (1:1000, Santa Cruz Biotechnologies.).

### RNA extraction, reverse transcription, and quantitative PCR

According to the standard protocol, total RNA was extracted from each group of cells using the TRIzol reagent kit (Invitrogen). Complementary DNA (cDNA) was synthesized using the PrimeScript™ RT reagent kit (Takara Bio, Inc., Otsu, Japan). Subsequently, mRNA expression was detected using the SYBR Premix Ex Taq™ reagent kit (Takara Bio) and the ABI 7900HT real-time PCR system (Applied Biosystems Life Technologies, CA, USA). Finally, the relative levels of mRNA in the cells were determined by applying the comparative threshold cycle (2 − ΔΔCt) method.

### Co-immunoprecipitation

Approximately 2 × 10^7^ cells were lysed using NP-40 buffer. Then, 20 μL of Protein A/G Sepharose beads (Santa Cruz, CA, USA) were added for pre-clearing, followed by centrifugation to collect the supernatant. The pre-cleared lysate was divided into input, negative control (IgG clone), and anti-MDH2 or anti-FSP1 rabbit polyclonal antibody groups. After adding the respective antibodies, the lysate was incubated with rotation at 4 °C for 12 h. Subsequently, 50 μL of protein A/G Sepharose beads were added to the lysate to capture the immune complexes, followed by 4 h of incubation. The complexes were then washed four times with NP-40 buffer by centrifugation at 3000 x g. The pellet was resuspended in 2x SDS loading buffer and boiled at 95 °C for 5 min to release the proteins. Finally, Western blot analysis was performed using anti-MDH2 or anti-FSP1 rabbit polyclonal antibodies.

### Animal Experiment

Male BALB/c-nu mice aged 6 weeks (Beijing Vital River Laboratory Animal Technology Co., Ltd.) were selected as the experimental subjects. All detailed experimental procedures were approved by the Institutional Animal Care and Utilization Committee of Fudan University Pudong Animal Experimental Center. 32 mice were randomly divided into 4 groups (*n* = 8) (786-O-KONC-Blank, 786-O-MDH2 KO1-Blank, 786-O-KONC-Sulfasalazine, 786-O-MDH2 KO1-Sulfasalazine). 786-O-KONC or 786-O-MDH2 KO1 cells (5 × 10^6^) suspended in 100 µl PBS were subcutaneously injected into the axilla of each nude mouse. After 10 days, different regimens were administered to the mice: solvent (100 μl 0.1 M NaOH + 100 μl saline) and sulfasalazine (200 mg/kg, orally, dissolved in 100 μl 0.1 M NaOH). Tumor diameters (L: longest diameter, S: shortest diameter) were measured with calipers every 3 days (tumor volume = L×S^2^/2). The growth curve of subcutaneous tumors was generated based on the measured tumor volumes. Euthanasia was performed using carbon dioxide anesthesia after 4–6 weeks. Observers and analysts are unaware of the grouping and medication situation of the animals.

### Immunohistochemistry

The tissues were embedded in paraffin and then each block was sectioned at a thickness of 3μm. The sections were incubated with 3% H_2_O_2_ in methanol to inhibit endogenous peroxidase, and then incubated with protein-blocking solution to block non-specific antigens. For immunostaining against MDH2, FSP1, and 4-hydroxy-2-nonenal (4-HNE), the sections were incubated with human MDH2 antibody, human FSP1 antibody, and mouse 4-HNE antibody. A secondary biotinylated anti-IgG antibody was used. Sections were visualized using horseradish peroxidase and 3-amino-9-ethylcarbozole as the substrate-chromogen (Dako). The nuclei were counterstained using hematoxylin. The immunostain-positive area was quantified with an image analysis system.

### Statistical analysis

All experiments were performed at least 3 times. SPSS software (version 19.0, IBM Corp., Armonk, NY, USA) was used for statistical analysis of all the experimental data. GraphPad Prism (version 7, GraphPad Software, La Jolla, CA, USA) was used to visualize the statistical results. All data are expressed as the mean ± standard deviation (mean ± sd) values. Statistical analysis of data from 2 groups was performed using a *t* test. Comparisons among multiple groups were performed by one-way ANOVA followed by the LSD-t test. *P* < 0.05 was considered to be significant.

### Supplementary information

WB original

Clinicopathologic characteristics of ccRCC patients

## Data Availability

The datasets generated and analyzed during the current study are available from the corresponding author on reasonable request.
