# Supplementary figures and images for: MDH2 regulates the sensitivity of clear cell renal cell carcinoma to ferroptosis through its interaction with FSP1
Source: Cell Death Discov. 2024 Aug 13;10:363. doi: 10.1038/s41420-024-02137-6 (PMC11322664; doi:10.1038/s41420-024-02137-6)

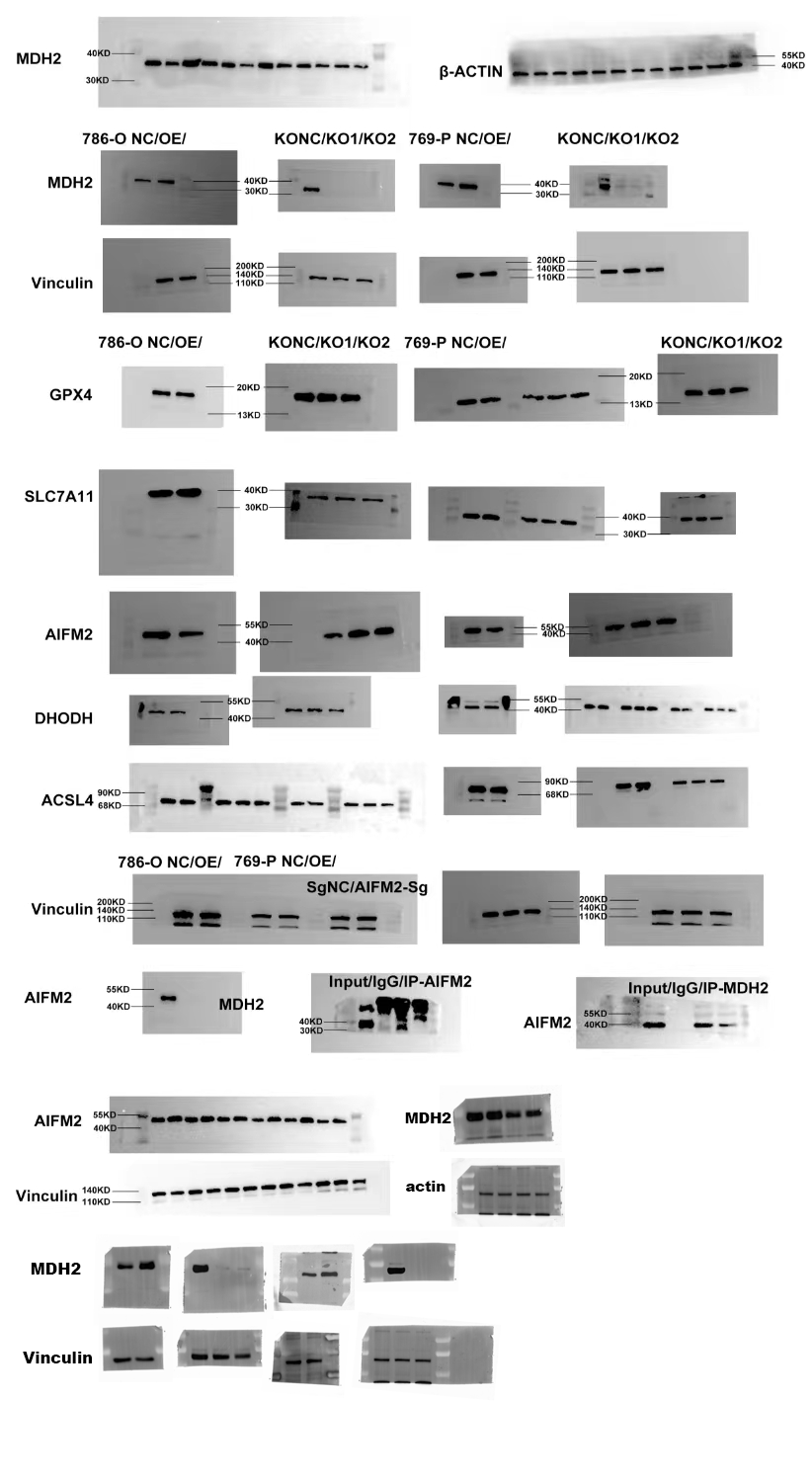

Supplement: Supplementary file 1 — WB original [file 41420_2024_2137_MOESM1_ESM.png]
