# Supplementary material for: MDH2 regulates the sensitivity of clear cell renal cell carcinoma to ferroptosis through its interaction with FSP1
Source: Cell Death Discov. 2024 Aug 13;10:363. doi: 10.1038/s41420-024-02137-6 (PMC11322664; doi:10.1038/s41420-024-02137-6)
Supplement: Supplementary file 2 — Clinicopathologic characteristics of ccRCC patients [file 41420_2024_2137_MOESM2_ESM.docx]

S Table1 Clinicopathologic characteristics of KIRC patients

| Characteristic |  | Patient |
| --- | --- | --- |
| All patient  Gender  Age（years)  Stage  T stage  N stage  M stage  Tumor size（cm） | Male  Female  ≤55  ＞55  Ⅰ+Ⅱ  Ⅲ+Ⅳ  T1+T2  T3+T4  N0  N1  M0  M1  ≤4  ＞4 | 85  37  48  14  71  29  56  26  59  23  62  43  42  21  64 |
